# The Association between D-Dimer and Prognosis in the Patients with Oral Cancer

**DOI:** 10.3390/dj8030084

**Published:** 2020-08-03

**Authors:** Kenji Yamagata, Satoshi Fukuzawa, Naomi Ishibashi-Kanno, Fumihiko Uchida, Toru Yanagawa, Hiroki Bukawa

**Affiliations:** Department of Oral and Maxillofacial Surgery, Institute of Clinical Medicine, Faculty of Medicine, University of Tsukuba, 1-1-1 Tennodai, Tsukuba, Ibaraki 305-8575, Japan; sfukuzawa3104@yahoo.co.jp (S.F.); greened_amethyst829@hotmail.com (N.I.-K.); uchiyamada1031@yahoo.co.jp (F.U.); ytony@md.tsukuba.ac.jp (T.Y.); bukawah-cuh@umin.ac.jp (H.B.)

**Keywords:** oral cancer (OC), D-dimer, overall survival (OS), management, neutrophil lymphocyte ratio (NLR), C-reactive protein (CRP)

## Abstract

D-dimer levels are reported to relate with tumor stage, prognosis, and lymph node involvement, as well as overall survival (OS) in patients with solid tumors. The purpose of this study was to investigate association between the value of D-dimer and the prognosis of oral cancer (OC). We designed a retrospective cohort study and enrolled a sample of patients who were diagnosed with OC and treated with surgery and/or radiotherapy. The predictor was the D-dimer and outcome variable was OS. Other variables included age, neutrocyte count, neutrophil lymphocyte ratio (NLR), C-reactive protein (CRP), and management. Differences in OS rate were analyzed by log-rank test. A Cox proportional hazards model was used to adjust for the effects of potential confounders. Differences with a P value less than 0.05 were considered statistically significant. In 88 patients with OC, D-dimer median value for the predicting OS was 0.7 µg/mL. There was a significant difference in OS when patients were stratified according to D-dimer, with an OS rate of 77.8% for patients with low D-dimer (<0.7), and 57.3% with high D-dimer (≥0.7) (*p* = 0.035). Univariate analyses revealed close correlations between OS and age, neutrocyte count, NLR, CRP, and D-dimer (<0.7 and ≥0.7). Cox multivariate analysis identified management (mainly surgery vs. radiotherapy) (HR 3.274, 95% CI 1.397–7.676; *p* = 0.006) as independent predictive factors for OS. There was a significant difference in OS when patients were stratified according to D-dimer with low (<0.7) and high D-dimer (≥0.7) (*p* = 0.035). Though, as a predictive factor, management was associated with OS.

## 1. Introduction

D-dimer is a stable end product of the degradation of cross-linked fibrin, which results from enhanced fibrin formation and fibrinolysis. Recently, it has been reported that increases in D-dimer levels have a correlation between malignant diseases. Moreover, some studies report that D-dimer levels are related to tumor stage, tumor prognosis, and lymph node metastasis, as well as overall survival (OS) in patients with lung cancer, breast cancer, gastric cancer, colon cancer, and gynecological cancer. Elevated D-dimer levels may influence multifactorial interactions between carcinoma growth and the hemostatic fibrinolytic system in malignancy [1,2,3,4].

The 8th edition of the American Joint Committee on Cancer (AJCC) staging in oral squamous cell carcinoma (OSCC) was published and two new parameters, namely depth of invasion (DOI) and extranodal extension (ENE), were included. The use of ENE in pN classification was reported as identifying patients with a worse prognosis. Furthermore, the use of lymph node ratio (LNR) improve the predictive capacity of the AJCC staging manual [5]. The ranges of D-dimer levels in head and neck (H&N) cancer patients differed from other malignancies. D-dimer levels in patients with the H&N cancer were much lower than those in patients with gynecological, esophageal, stomach, colorectal, or lung cancer because H&N cancers have lower metastatic rates and a more favorable prognosis than these malignancies [6]. To the best of our knowledges, there was only one report that high D-dimer levels were associated with OS and an increased risk of mortality in nasopharyngeal cancer patients [7]. The purpose of this study was to investigate the association between the value of D-dimer and the prognosis in patients with OC.

## 2. Materials and Methods

### 2.1. Study Design and Sample

To address the research purpose, we designed and implemented a retrospective cohort study. The study population was composed of all patients presenting for evaluation and management subjects from patients who were diagnosed with OC and received treatment with surgery and/or radiotherapy for 3 years between 2015 to 2018 at the Department of Oral and Maxillofacial Surgery, University of Tsukuba Hospital (Ibaraki, Japan). Of about 155 oral cancer patients, excluding no surgical and/or radiotherapy and no d-dimer evaluation at pretreatment, 88 patients were included in this study which took place more than 1 year after treatment. Serum D-dimer concentration was evaluated pretreatment and during treatment, while venous ultrasonography (US) of the lower extremities was performed by an experienced sonographer to establish the incidence and location of deep vein thrombosis (DVT) for the patients D-dimer > 1.0 μg/mL according to rules of our hospital. The iliac, femoral, great saphenous, popliteal, peroneal, post-tibial, and soleal veins were evaluated bilaterally [8]. Surgical patients with surgical time more than 2 h received mechanical thromboprophylaxis, such as compression stockings and intermittent pneumatic compression during surgery. Cancer was staged according to the 2017 Union for International Cancer Control (UICC) categories. This study was conducted in accordance with the Declaration of Helsinki and was approved by the Institutional Review Board of University of Tsukuba Hospital (No. R01-270, 31 January 2020). Informed consent was waived due to the retrospective nature of the study.

### 2.2. Study Variables

The primary predictor variable of this study was pre-treatment D-dimer value. The patients were divided into binary subgroups using the median value as the cut-off point, which determined that the best cut-off value for D-dimer was 0.7 µg/mL. The D-dimer level of cancer patients which served as an indicator of prognosis was reported as 0.7 μg/mL in lung cancer [6]. The primary outcome variable was OS, and the other variables were age, management, and blood laboratory results, namely neutrophil count, neutrophil lymphocyte ratio (NLR), and C-reactive protein (CRP).

### 2.3. Data Analyses

Patients were divided into high- or low-risk subgroups using the median values for the D-dimer, and differences between the subgroups were analyzed for significance. Survival curves were calculated by the Kaplan–Meier method, and differences in OS rate was analyzed by log-rank test. OS was calculated from the date of first diagnosis to death from any cause. The cut-off date for surviving patients was December 2019 and median (IQR) time point of OS analyzed was 28.2 (16.9–37.1) months. Subgroups were compared by the Mann–Whitney U test and χ^2^ test. A Cox proportional hazards model was used to adjust for the effects of potential confounders. All statistical analyses were performed with the SPSS software version 25 for Macintosh (SPSS, Chicago, IL, USA). Differences with a *p* value less than 0.05 were considered statistically significant.

## 3. Results

### 3.1. Patient Characteristics

We retrospectively reviewed 88 patients who were diagnosed with OC. Based on D-dimer median value, there were 28 patients with low D-dimer (<0.7) and 60 with high D-dimer (≥0.7). In terms of gender, there were 56 men and 32 women with a median age of 72.5 years (range 34–93). The most common primary tumor sites were tongue (*n* = 27) and mandibular gingiva (*n* = 25). The association with study variables versus D-dimer are presented in Table 1 and Table 2. The median D-dimer was 0.7 μg/mL (range 0.4–12.2 μg/mL). The variables associated with D-dimer were smoking, age, and CRP. There were 22(78.6%) patients with low D-dimer (<0.7) and 34 (56.7%) with high D-dimer (≥0.7) absent smoking. There was a significant difference regarding the absence of smoking, pre and present (*p* = 0.019). The D-dimer was associated with a median age of 65.5 years of D-dimer (<0.7) and 76.0 years D-dimer (≥0.7) (*p* < 0.0001), while CRP 0.40 mg/µL of D-dimer (<0.7) and 0.15 mg/µL of D-dimer (≥0.7) (*p* = 0.002).

### 3.2. Clinical Factors and Survival

**Figure 1:**
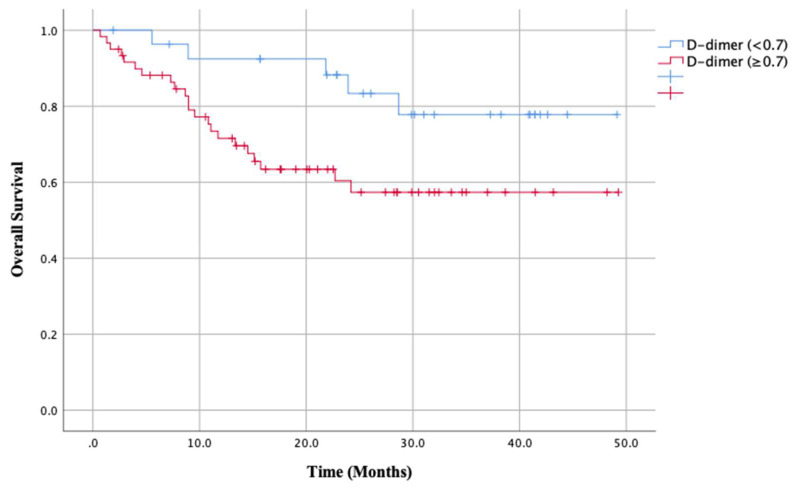
Kaplan–Meier analysis according to D-dimer with a median of 0.7. When patients were stratified by D-dimer, the overall survival rate was 77.8% for patients with low D-dimer (<0.7) and 57.3% for patients with high D-dimer (≥0.7). This difference was significant (*p* = 0.035).

The association with study variables versus overall survival were presented in Table 3. The variables associated with OS was only management (*p* < 0.0001). Surgical treatment with only surgery, surgery and radiotherapy, surgery and chemoradiotherapy were performed in 70 patients with OS 72.6% and radiotherapy and chemoradiotherapy in 18 patients with OS 22.3%. There was a significant difference in OS between main treatment of surgery and radiotherapy (*p* < 0.0001). There were no significant differences in OS when patients were stratified by stage classification (OS rates: I 75%, II 72.6%, III 50.0%, IVA 62.4% and IVB 41.7%). In contrast, there were significant differences in the primary outcome variable of OS when patients were stratified according to the primary predictor variable (D-dimer (<0.7 vs. ≥0.7)), with a rate of 77.8% for patients with low D-dimer (<0.7) and 57.3% for patients with high D-dimer (≥0.7) (*p* = 0.035; Figure 1).

### 3.3. Cox Multivariate Regression Analysis

Univariate analyses showed that OS was significantly associated with D-dimer (<0.7 vs. ≥0.7), with a hazards ratio (HR) of 2.744 and 95% confidence interval (CI) of 1.034–7.281 (*p* = 0.043). We also found significant associations between OS and age (HR 1.035, 95% CI 1.001-1.069; *p* = 0.042), neutrocyte count (HR 1.297, 95% CI 1.061–1.585; *p* = 0.011), NLR (HR 1.172, 95% CI 1.007–1.364: *p* = 0.041), CRP (HR 1.174, 95% CI 1.036–1.329: *p* = 0.012), and management (mainly surgery vs. radiotherapy; HR 4.972, 95% CI 2.282–10.834; *p* = 0.0002). Details are shown in Table 4.

Cox multivariate analysis of the parameters selected by univariate analysis identified one independent predictive factor for OS, namely management (surgery vs. radiotherapy; HR 3.274, 95% CI 1.397–7.676: *p* = 0.006). This result indicates that the management (surgery vs. radiotherapy) is a better prognostic marker than the D-dimer (<0.7 vs. ≥0.7).

## 4. Discussion

D-dimer level is associated with risk of venous thromboembolism (VTE) in cancer patients. Guidelines for the management of VTE provided by the American Society of Hematology in 2018 recommended, for patients at low VTE risk, using D-dimer as the initial test reduces the need for diagnostic imaging. For pulmonary embolism diagnosis, ventilation-perfusion scanning and computed tomography (CT) pulmonary angiography are the most validated tests, whereas lower or upper extremity DVT diagnosis uses US [9]. D-dimer, a stable end product of the degradation of cross-linked fibrin, results from enhanced fibrin formation and fibrinolysis. In the H&N cancer surgery, D-dimer is used in the diagnosis of VTE and reported association of free flap venous thrombosis. For patients with preoperative D-dimer < 0.4 μg/mL, the likelihood of venous thrombosis was greater than for patients with D-dimer ≥ 0.4 μg/mL. The anticoagulation and coagulation systems are always in a dynamic balance. Therefore, the preoperative higher D-dimer values represented a more effective anticoagulation system in patients with free flap success [10]. On the other hand, D-dimer level was independent of gender but was affected by the age of the patient [6]. In our study, there was no significant difference in gender but in age between D-dimer (<0.7 and ≥0.7). Age adjustment is desired to decide the optimal cut off value with large samples in a future study. Moreover, there was a significant difference in CRP between D-dimer (<0.7 and ≥0.7). A previous study reported that the correlation between CRP and D-dimer, and a significant elevation among patients with DVT [11].

Recently, it has been reported that increases in D-dimer levels are correlated with malignant diseases. D-dimer level is associated with risk of VTE and DVT in cancer patients. Most cancer patients have abnormal D-dimer levels based on the normal reference range. The D-dimer range of H&N cancer was 0.22, 0.44, and 2.19 (median, 5th, 95th). Various cancer patients with high initial D-dimer were shown a tendency of poor prognosis in survival rate [6]. Some studies report that D-dimer levels have a relation to tumor stage, prognosis, and lymph node metastasis, as well as OS in patients with solid tumors, such as lung, breast, gastric, colon cancers [1,2,3,4]. Although there was a significant difference in OS when patients were stratified according to D-dimer with low D-dimer (<0.7) and high D-dimer (≥0.7) (*p* = 0.035) in our study, not selected as a predictive factor in multivariate survival analysis. It was reported that H&N cancers have lower metastatic rates and a more favorable prognosis than other malignancies and the D-dimer level was lower than other ones [6]. Our OC cases may correlate with these previous results of lower metastatic rates and a favorable prognosis than other cancers.

To the best of our knowledges, there was no report about indicator of poor prognosis with optimal cut-off value in OC. Yu et al. reported tumor specific D-dimer concentration according to various cancers. Cancer patients with high initial D-dimer reported to be shown a tendency of poor prognosis in survival rate. The initial D-dimer level of lung cancer patients which served as an indicator of prognosis was reported as 0.7 μg/mL [6]. Altiay et al. reported a cut-off value (≤0.65 and >0.65) for the optimal prediction of survival for lung cancer [12]. Moreover, the median value of D-dimer was 0.7 μg/mL in our study. Therefore, we defined the cut-off value ≥ 0.7 μg/mL with predicting poor prognosis from lung cancer reports and our median value. On the other hand, 0.8 μg/mL which was 3rd quartile values as the optimal cut-offs, high D-dimer levels were associated with poor OS in nasopharyngeal cancer patients [7]. The D-dimer levels predicting poor prognosis in gastrointestinal cancer of a meta-analysis reported 0.6 μg/mL [2]. Our initial D-dimer level of median with 0.7 μg/mL nearly corresponded with these previous reports. However, our sample size is small, and a further study will be desired in a large cohort to make a conclusion regarding the defined D-dimer value which can serve as an indicator of poor prognosis in OC.

In univariate analysis, there were significant differences in neutrocyte count, NLR and CRP with OS. The systemic inflammatory response has been shown to play an important role in cancer progression. The CRP as a sensitive measure of the systemic inflammatory response reported an independent prognostic value [13]. Recently, CRP and albumin with the Glasgow prognostic score (GPS) reported an independent prognostic value in patients with cancer [14]. Moreover, NLR was indicated which reduced the survival rate and associates with cancer prognosis [15]. Our results are correlated with these reports about the association with the systemic inflammatory response markers and cancer prognosis.

The OS of management with surgery was 72.6% and radiotherapy was 22.3%. Although the stage had no significant difference with OS, only management had a significant difference in multivariate analysis. This may be a selection bias according to unresectable cancer and/or non-adaptation for surgery because of bad general conditions in favor of surgery or radiotherapy. A limitation of this study is the retrospective nature, the small sample, and short observation periods of patients with OC which warrants further validation in a large cohort. Therefore, prospective and multicentral studies are needed to find an accurate relation between D-dimer and the outcome of patients with OC.

## 5. Conclusions

There was a significant difference in OS when patients were stratified according to D-dimer value with low D-dimer (<0.7) and high D-dimer (≥0.7) (*p* = 0.035). Though, as a predictive factor, only management was associated with OS.

## Figures and Tables

**Table 1:** Study variables versus D-dimer.

Variable	D-Dimer (<0.7) (*n* = 28)	D-Dimer (≥0.7) (*n* = 60)	*p* Value
**Gender**			0.574
Male	19 (67.9%)	37 (61.7%)	
Female	9 (32.1%)	23 (38.3%)	
**Site**			0.962
Tongue	9 (32.1%)	18 (30.0%)	
Lower gingiva	8 (28.6%)	17 (28.3%)	
Buccal mucosa	3 (10.7%)	9 (15.0%)	
Upper gingiva	2 (7.1%)	6 (10.0%)	
Floor of mouth	3 (10.7%)	3 (5.0%)	
Maxillary sinus	1 (3.6%)	3 (5.0%)	
Others	2 (7.1%)	4 (6.7%)	
**T stage**			0.501
T1	7 (25%)	7 (11.7%)	
T2	7 (25%)	20 (30.0%)	
T3	1 (3.6%)	5 (8.3%)	
T4a	9 (32.1%)	21 (35.0%)	
T4b	4 (14.3%)	7 (11.6%)	
**N stage**			0.402
N0	20 (71.4%)	31 (51.7%)	
N1	3 (10.7%)	9 (15.0%)	
N2b	5 (17.9%)	16 (26.7%)	
N2c	0 (0%)	3 (5.0%)	
N3	0 (0%)	1 (1.7%)	
**Stage**			0.456
I	7 (25%)	6 (10.0%)	
II	6 (21.4%)	14 (23.3%)	
III	1 (3.6%)	3 (5.0%)	
IVA	11 (39.3%)	31 (51.7%)	
IVB	3 (10.7%)	6 (10.0%)	
**Management ^a^**			0.157
S	16 (57.1%)	34 (56.7%)	
S+RT	4 (14.3%)	4 (6.7%)	
S+CRT	6 (21.4%)	6 (10.0%)	
RT	1 (3.6%)	9(15.0%)	
RT+C	1 (3.6%)	7 (11.7%)	
**Pathological diagnosis ^b^**			0.093
SCC	25 (89.3%)	59 (98.3%)	
ACC	2 (7.1%)	0 (0%)	
Others	1 (3.6%)	1 (1.7%)	
**VTE ^c^**			0.094
Absent	27 (96.4%)	49 (81.7%)	
Present	1 (3.6%)	11 (18.3%)	
**Smoking**			0.019 *
Absent	22 (78.6%)	34 (56.7%)	
Pre	1 (3.6%)	18 (30.0%)	
Present	5 (17.9%)	8 (13.3%)	
**Alcohol**			0.299
Absent	20 (71.4%)	36 (60.0%)	
Present	8 (28.6%)	24 (40.0%)	

^a^ Management: S, surgery; RT, radiotherapy; C, chemotherapy; CRT, chemoradiotherapy. ^b^ Pathological diagnosis: SCC, squamous cell carcinoma; ACC, adenoidocystic carcinoma. ^c^ VTE, venous thromboembolism. * *p* < 0.05. N stage: N0 vs. N2b, 2c, 3 (*p* = 0.080). Management: S, S + RT, S + CRT vs. others (*p* = 0.046 *).

**Table 2:** Study variables versus D-dimer.

Variable Median (Range)	D-Dimer (<0.7) (*n* = 28)	D-Dimer (≥0.7) (*n* = 60)	*p* Value
Age (Years)	65.5 (34~84)	76.0 (39~93)	<0.0001 **
BMI (Kg/m^2^)	21.52 (16.81~29.48)	22.26 (14.29~32.65)	0.610
Caprini Score	6 (4~8)	6 (4~8)	0.064
Neutrocyte count (×10^3^/µL)	4.129 (1.995~6.845)	4.513 (1.666~8.880)	0.182
CRP (mg/µL)	0.40 (0~0.60)	0.15 (0.03~10.92)	0.002 **
PLT count (×10^3^/µL)	216.50 (164.0~384.0)	226.5 (68.0~454.0)	0.750
Lymphocyte count (×10^3^/µL)	1.653 (0.630~2.843)	1.570 (0.529~3.081)	0.979
Monocyte count (×10^3^/µL)	0.358 (0.168~0.820)	0.384 (0.122~0.792)	0.232
LMR	4.72 (1.66~9.85)	3.98 (1.461~19.263)	0.216
NLR	2.34 (1.38~6.99)	2.91 (0.96~12.22)	0.173
PLR	150.8 (82.7~344.5)	148.5 (70.6~319.6)	0.795

BMI: body mass index, CRP: C-reactive protein, PLT: platelet, LMR: lymphocyte monocyte ratio, NLR: neutrocyte lymphocyte ratio, PLR: platelet lymphocyte ratio, ** *p* < 0.01.

**Table 3:** Study variables versus overall survival.

Variable	No of Patients (%)	Overall Survival (%)	*p* Value
**Gender**			0.842
Male	56 (63.6)	61.8	
Female	32 (36.4)	67.3	
**Site**			0.588
Tongue	27 (30.7)	59.6	
Lower gingiva	25 (28.4)	76.1	
Buccal mucosa	12 (13.6)	46.9	
Upper gingiva	8 (9.1)	75.0	
Floor of mouth	6 (6.8)	62.5	
Maxillary sinus	4 (4.5)	50.0	
Others	6 (6.8)	80.0	
**T stage**			0.270
T1	14 (15.9)	68.2	
T2	27 (30.7)	77.2	
T3	6 (6.8)	55.6	
T4a	30 (34.1)	58.3	
T4b	11 (12.5)	39.8	
**N stage**			0.899
N0	51 (58.0)	65.2	
N1	12 (13.6)	73.3	
N2b	21 (23.9)	55.1	
N2c	3 (3.4)	66.7	
N3	1 (1.1)	100	
**Stage**			0.364
I	13 (14.8)	75.0	
II	20 (22.7)	72.6	
III	5 (5.7)	50.0	
IVA	42 (4.8)	62.4	
IVB	9 (10.2)	41.7	
**Management ^a^**			<0.0001 **
S	50 (56.8)	90.0	
S+RT	8 (9.1)	37.5	
S+CRT	12 (13.6)	40.0	
R	10 (11.4)	28.1	
R+C	8 (9.1)	21.9	
**Pathological diagnosis ^b^**			0.719
SCC	84 (95.5)	63.3	
ACC	2 (2.3)	100	
Others	2 (2.3)	50.0	
**VTE ^c^**			0.387
Absent	76 (86.4)	71.1	
Present	12 (13.6)	58.3	
**Smoking**			0.922
Absent	56 (63.6)	63.1	
Pre	19 (21.6)	73.0	
Present	13 (14.8)	61.5	
**Alcohol**			0.345
Absent	56 (63.6)	65.9	
Present	32 (36.4)	59.6	

^a^ Management: S, surgery; RT, radiotherapy; C, chemotherapy; CRT, chemoradiotherapy.^b^ Pathological diagnosis: SCC, squamous cell carcinoma; ACC, adenoidocystic carcinoma.^c^ VTE, venous thromboembolism, ** *p* < 0.01. Management: S, S + RT, S + CRT vs. R, R+C; OS 72.6% vs. 22.3% (*p* < 0.0001 **).

**Table 4:** Prognostic factors for overall survival ^a^.

Factor	HR	95% CI	*p* Value
**Univariate analysis**			
Age	1.035	1.001–1.069	0.042 *
BMI	0.968	0.860–1.089	0.377
Smoking (Present vs. Pre, Absent)	1.161	0.438–3.076	0.764
Caprini Score	0.877	0.630–1.222	0.439
Stage (I, II vs. II, IV)	0.443	0.179–1.098	0.079
PLT	1.001	0.996–1.006	0.767
Neutrocyte count	1.297	1.061–1.585	0.011 *
Lymphocyte count	0.625	0.313–1.413	0.289
Monocyte count	6.610	0.597–73.151	0.124
NLR	1.172	1.007–1.364	0.041 *
LMR	0.855	0.693–1.055	0.144
PLR	1.002	0.996–1.007	0.583
CRP	1.174	1.036–1.329	0.012 *
D-dimer (<0.7 vs. ≥0.7)	2.744	1.034–7.281	0.043 *
Management (Mainly S vs. R)	4.972	2.282–10.834	0.0002 *
**Multivariate survival analysis**			
Age	1.027	0.984–1.072	0.222
Neutrocyte count	1.247	0.957–1.625	0.101
CRP	0.969	0.821–1.1441	0.713
D-dimer (<0.7 vs. ≥0.7)	1.332	0.440–4.032	0.612
Management (Mainly S vs. R)	3.274	1.397–7.676	0.006 *

HR, hazards ratio; 95% CI, 95% confidence interval; BMI, body mass index; PLT, platelet; NLR, neutrocyte lymphocyte ratio; LMR, lymphocyte monocyte ratio; PLR, platelet lymphocyte ratio; CRP, C reactive protein. ^a^ Evaluated by Cox proportional hazards regression, * *p* < 0.05.
